# Gender Inequality and Late-Life Cognition

**DOI:** 10.1093/geroni/igaa057.2278

**Published:** 2020-12-16

**Authors:** Jinkook Lee, Marco Angrisani, Urvashi Jain

**Affiliations:** 1 University of Southern California, Los Angeles, California, United States; 2 University of South Alabama, Mobile, Alabama, United States

## Abstract

Indian women’s disadvantage in cognition has been documented in several studies, additionally noting geographic variations. The gender gap in late-life cognition could be a manifestation of gender discrimination. Using national data from LASI-DAD together with information drawn from administrative data, we construct a state-level composite index of gender inequality, following the UNDP definition. We investigate and find strong evidence that cross-state differences in gender inequality are significantly associated with the gender gap in cognition. Women in the most discriminating state (Bihar) perform significantly worse than men (-0.21 s.d.), after controlling for key risk factors such as age and education. The gender gap in the least discriminating state (Kerala) is much smaller (-0.10 s.d.). We also find that gender inequality is strongly associated with education, early marriage, labor force participation, and social activities. This has important implications for public health policy aimed at reducing the risk of cognitive impairment and dementia.

